# Supplementary material for: A potential histone-chaperone activity for the MIER1 histone deacetylase complex
Source: Nucleic Acids Res. 2023 Apr 26;51(12):6006–19. doi: 10.1093/nar/gkad294 (PMC10325919; doi:10.1093/nar/gkad294)
Supplement: gkad294_Supplemental_Files [file gkad294_supplemental_files.zip › SUPPLEMENTARY MATERIAL.pdf]

SUPPLEMENTARY MATERIAL

An unexpected histone-binding activity for the MIER1 histone deacetylase complex suggests a potential chaperone function.

Siyu Wang[1], Louise Fairall[1], Trong Khoa Pham[2,3], Timothy J Ragan[1], Dipti Vashi[1], Mark O. Collins[2,3], Cyril Dominguez[1] and John W.R. Schwabe[1]*

1 Institute for Structural and Chemical Biology & Department of Molecular and Cell Biology, University of Leicester, Leicester. LE1 7RH. UK.
2 School of Biosciences, University of Sheffield, Sheffield. S10 2TN. UK.
3 biOMICS facility, Mass Spectrometry Centre, University of Sheffield, Sheffield. S10 2TN. UK.
* Corresponding author: john.schwabe@leicester.ac.uk

a)

```
MIER1(HUMAN)   ------------------MAEPS-----------------------------VESSSPGG      13
MIER2(HUMAN)   ------------------MAEASSL-------------GRQSPRVVSCLEHSLCPGEPGL      29
MIER3(HUMAN)   ------------------MAEAS-----------------------------FGSSSPVG      13
MIER1(MOUSE)   ------------------MAEPS-----------------------------VESSSPGG      13
MIER1(CHICK)   ------------------MAEPS-----------------------------VESSSPGG      13
MIER1(XENLA)   ------------------MAEPS-----------------------------LRTASPGG      13
MIER1(DANIO)   ------------------MAEPS-----------------------------LSASGSEG      13
A1Z6Z7(DROME)  MMEFGKSSDHSQHSASEASADSGSLANSDLAVTPTKRERRKSPATS------SDNNSTSV      54

                         Region1
MIER1(HUMAN)   S------ATSDDHEFDPSADMLVHDFDDERTLEEEEMMEGETN--FSSEIEDLAREGDMP      65
MIER2(HUMAN)   QTTAVVSMGSGDHQFNL-AEILSQNYSVRGECEEASRCPDKPK--EELEKDFISQSNDMP      86
MIER3(HUMAN)   S------LSSEDHDFDPTAEMLVHDYDDERTLEEEEMMDEGKN--FSSEIEDLEKEGTMP      65
MIER1(MOUSE)   S------ATSEDHEFDPSADMLVHDFDDERTLEEEEMMEGETN--FSSEIEDLAREGDMP      65
MIER1(CHICK)   S------ATSDDHEFDPSADMLVHDFDDERTLEEEEMMEGERN--FNSEIEDLNRESDMP      65
MIER1(XENLA)   S------AASDDHEFEPSADMLVHEFDDEQTLEEEEMLEGEVN--FTSEIEHLERESEMP      65
MIER1(DANIO)   A---HAVVQDEDKDFDPSADMLVHDFDDEQTLEEQEKLEGETN--FTNEIDDLTREGEMP      68
A1Z6Z7(DROME)  QPPMSPSSSVADTTFEPTIDMMVNDFDDEATLNEEEALADMEAHSAEDEIATLREESEMP     114

                Region2
MIER1(HUMAN)   IHELLSLYGYGSTVRLPEEDEE--EEEEEEEGE-------DDEDADNDDNSGCSGENKEE     116
MIER2(HUMAN)   FDELLALYGYEASDPISDRESEGGD-----VAP-------NLPDM--------TLDKEQI     126
MIER3(HUMAN)   LEDLLAFYGYEPTIPAVANSSANSSPSELA--D-------ELPDM--------TLDKEEI     108
MIER1(MOUSE)   IHELLSLYGYDSTVRLPEEEEE--E-EEEEEGE-------DDEDADNDDNSGCSGENKEE     115
MIER1(CHICK)   IQELLSLYGYDGTIPLQEDDDDEDEEEEEEEGE-------DDDDVDNDDNSGCSGENKEE     118
MIER1(XENLA)   IDELLRLYGYGSTVPLPGEEE-----------E-------DEEDMDNDCNSGCSGEIKDE     107
MIER1(DANIO)   IEELLKLYGYSSGASPEEEEEDVEEE-------------------DSTENACSKVEEKP     708
A1Z6Z7(DROME)  IEELLAKYGGTAASPAMSSSNRSGSSSRRARRATKRQYQELDTEMAHTSTST-SSSTSQL     173

                          Region3
MIER1(HUMAN)   NIKDSSGQ-EDETQSSNDDPSQ-SVASQDA-QEIIR-P--     150
MIER2(HUMAN)   AKDLLSGEEEEETQSSADDLTP-SVTSHEA-SDLFPNR--     162
MIER3(HUMAN)   AKDLLSGD-DEETQSSADDLTP-SVTSHET-SDFFPRP--     143
MIER1(MOUSE)   NIKDSSGQ-EDETQSSNDDPSQ-SVTSQDA-QEIIR-P--     149
MIER1(CHICK)   TIKDSSGQ-EDDTQSSNDDPAP-SVASQDP-QELIR-P--     152
MIER1(XENLA)   AIKDSSGQ-EDETQSSNDDPTP-SFTCRDV-REVIR-P--     141
MIER1(DANIO)   ELSDQE-E-DEDVQSSGEEPPS-GSVSHST-ALLIC-S--     141
A1Z6Z7(DROME)  EKHDGIDQ-EEEPKEEADKLAS*SVDTEDA*HLLDLYPDESF   232
```

b)

```
                                   Region1
MIER1(WT)   MAEPSVESSSPGGSATSDDHEFDPSADMLVHDFDDERTLEEEEMMEGETNFSSEIEDLAR
MIER1(D1)   MAEPSVESSSPGGSAT-------PSADMLVHDFDDERTLEEEEMMEGETNFSSEIEDLAR
MIER1(D2)   MAEPSVESSSPGGSATSDDHEFDPSADMLVHDFDDERTLEEEEMMEGETNFSSEIEDLAR
MIER1(D3)   MAEPSVESSSPGGSATSDDHEFDPSADMLVHDFDDERTLEEEEMMEGETNFSSEIEDLAR
MIER1(S1)   MAEPSVESSSPGGSATHDFDEDSPSADMLVHDFDDERTLEEEEMMEGETNFSSEIEDLAR
MIER1(S2)   MAEPSVESSSPGGSATSDDHEFDPSADMLVHDFDDERTLEEEEMMEGETNFSSEIEDLAR
            ****************       *************************************

                   Region2
MIER1(WT)   EGDMPIHELLSLYGYGSTVRLPEEDEEEEEEEEEGEDDEDADNDDNSGCSGENKEENIKD
MIER1(D1)   EGDMPIHELLSLYGYGSTVRLPEEDEEEEEEEEEGEDDEDADNDDNSGCSGENKEENIKD
MIER1(D2)   EGD------------GSTVRLPEEDEEEEEEEEEGEDDEDADNDDNSGCSGENKEENIKD
MIER1(D3)   EGDMPIHELLSLYGYGSTVRLPEEDEEEEEEEEEGEDDEDADNDDNSGCSGENKEE----
MIER1(S1)   EGDMPIHELLSLYGYGSTVRLPEEDEEEEEEEEEGEDDEDADNDDNSGCSGENKEENIKD
MIER1(S2)   EGDLSLYGYHELIPMGSTVRLPEEDEEEEEEEEEGEDDEDADNDDNSGCSGENKEENIKD
            ***            *****************************************

                         Region3
MIER1(WT)   SSGQEDETQSSNDDPSQSVASQDAQEIIRPRRCKYFDTNSEVEEESEEDEDYIPSED
MIER1(D1)   SSGQEDETQSSNDDPSQSVASQDAQEIIRPRRCKYFDTNSEVEEESEEDEDYIPSED
MIER1(D2)   SSGQEDETQSSNDDPSQSVASQDAQEIIRPRRCKYFDTNSEVEEESEEDEDYIPSED
MIER1(D3)   ----------------------------RPRRCKYFDTNSEVEEESEEDEDYIPSED
MIER1(S1)   SSGQEDETQSSNDDPSQSVASQDAQEIIRPRRCKYFDTNSEVEEESEEDEDYIPSED
MIER1(S2)   SSGQEDETQSSNDDPSQSVASQDAQEIIRPRRCKYFDTNSEVEEESEEDEDYIPSED
                                        *****************************
```

Supplementary Figure 1

a) Sequence alignment of amino terminus of MIER1, MIER2 and MIER3 and MIER proteins from model organisms. Residues are coloured by amino acid properties. Conserved regions 1, 2 and 3 are indicated by grey boxes.

b) Sequences of MIER1 (aa:1-177) showing details of the deleted and scrambled regions (D1, D2, D3, S1 and S2).

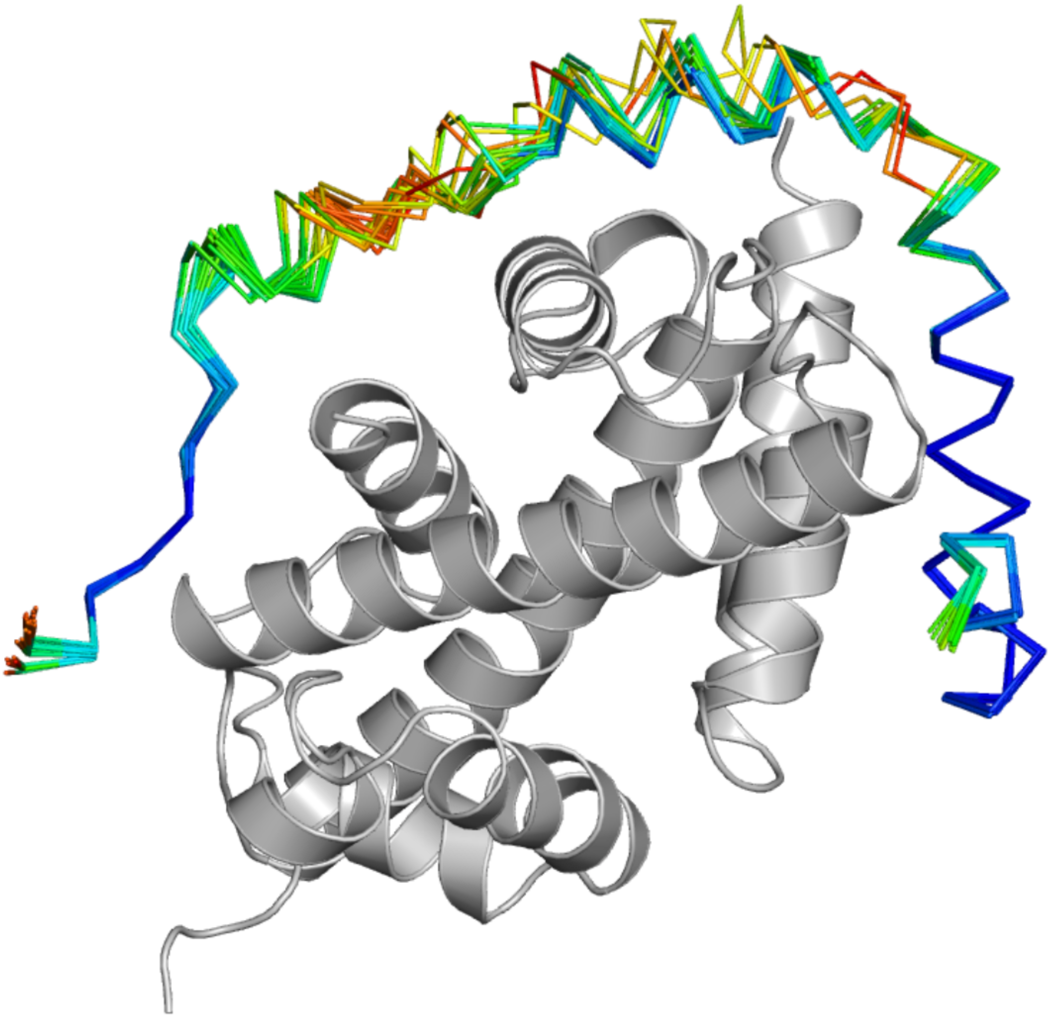

Supplementary Figure 2

Comparison of the 25 models generate by Alphafold2 Multimer predicting the interaction of MIER1(aa:17-75) (coloured by prediction confidence – blue = highest) with the H2A:H2B dimer (grey cartoon).

a)

```
                          Region1
MIER1(WT)      MAEPSVESSSPGGSATSDDHEFDPSADMLVHDFDDERTLEEEMMEGETNFSSEIEDLAR
MIER1(Mut1)    MAEPSVESSSPGGSATSDDHEFDPSADMLVHDFDDERTLEEEMMEGETNFSSEIEDLAR
MIER1(Mut2)    MAEPSVESSSPGGSATSDDHEADPSADAAVHDFDDERTLEEEMMEGETNFSSEIEDLAR
MIER1(Mut3)    MAEPSVESSSPGGSATSDDHEFDPSADMLVHDFDDERTLEEEMMEGETNFSSEIEDLAR

                    Region2
MIER1(WT)      EGDMPIHELLSLYGYGSTVRLPEEDEEEEEEEEEGEDDEDADNDDNSGCSGENKEENIKD
MIER1(Mut1)    EGDMPIHELLAAYGYGSTVRLPEEDEEEEEEEEEGEDDEDADNDDNSGCSGENKEENIKD
MIER1(Mut2)    EGDMPIHELLSLYGYGSTVRLPEEDEEEEEEEEEGEDDEDADNDDNSGCSGENKEENIKD
MIER1(Mut3)    EGDMPAHEAASLYGYGSTVRLPEEDEEEEEEEEEGEDDEDADNDDNSGCSGENKEENIKD

                         Region3
MIER1(WT)      SSGQEDETQSSNDDPSQSVASQDAQEIIRPRRCKYFDTNSEVEEESEEDEDYIPSED
MIER1(Mut1)    SSGQEDETQSSNDDPSQSVASQDAQEIIRPRRCKYFDTNSEVEEESEEDEDYIPSED
MIER1(Mut2)    SSGQEDETQSSNDDPSQSVASQDAQEIIRPRRCKYFDTNSEVEEESEEDEDYIPSED
MIER1(Mut3)    SSGQEDETQSSNDDPSQSVASQDAQEIIRPRRCKYFDTNSEVEEESEEDEDYIPSED
```

b)

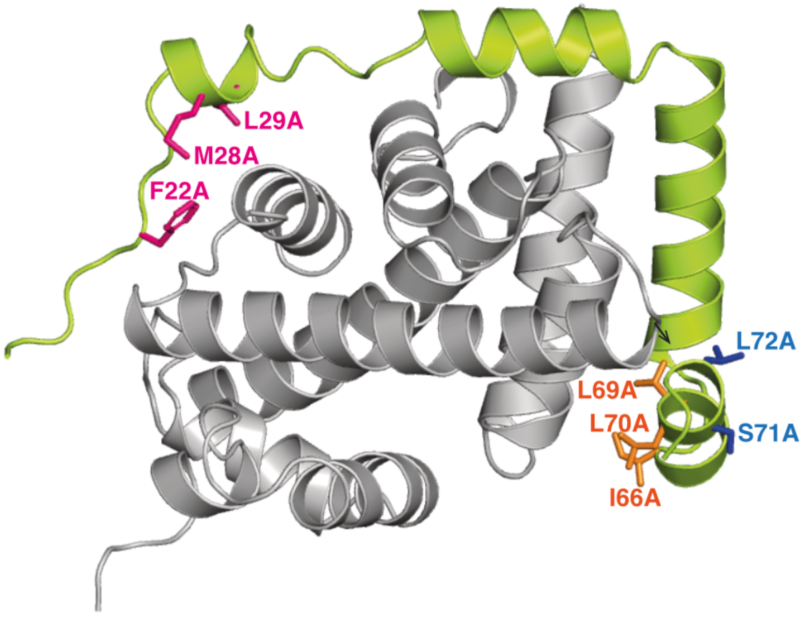

Supplementary Figure 3

a) Sequence alignment of amino terminus of MIER1(WT) showing the 3 sets of mutations to support the Alphafold2 model.

b) Location of the sets of mutations in a Alphafold2 model of the MIER1:H2A:H2B complex.

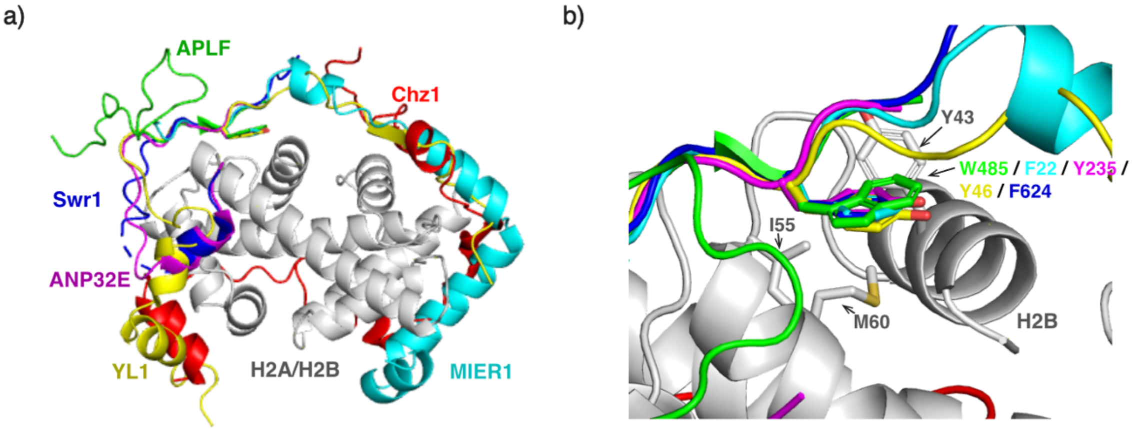

c)

PDB ID	Protein	Interface Å^2
Alpha Fold	MIER1	2005.3
2JSS	CHZ1	1812.8
4M6B	SWR1	974.7
4CAY	ANP32E	1062.4
5FUG	YL1	1824.5
6YN1	APLF	1102.2

Supplementary Figure 4

a) Comparison of the Alphafold2 multimer model of MIER1(aa:17-75), with Chz1 (PDB: 2JSS), Swr1 (PDB: 4M6B), ANP32E (PDB: 4CAY), YL1 (PDB:5FUG), APLF (PDB: 6YN1) all bound to a histone H2A:H2B dimer.

b) Detail of the shared non-polar interaction with the H2A:H2B dimer – coloured according to panel a).

c) Comparison the solvent excluded surface areas of the various complexes calculated by PISA (27).

```
hsMIER1   17-SDDHEFDPSADMLVHDFDDERTLEEEEMMEGETN--FSSEIEDLAREGDMPIHELLSLYGY-75
dmA1Z6Z7  64-VADTTFEPTIDMMVNDFDDEATLNEEEALADMEAHSAEDEIATLREESEMPIEELLAKYGG-124
               *  *:*: **:*:***** **:*** : .      ..**  * .*.:***.***: **
```

```
hsMIER1   17-SDDHEFDPSADMLVHDFDDERTLEEEEMMEGETNFSSEIEDLAREGDMPIHELLSLYGY-75
ceP91437  11-TDEEDYVDDN---DNGEDYEATIEEEELLD-EGDYEDELKDLEDEGEMSIEELQRKYGY-65
             :*:.::  .     :. * * *:****::: * ::..*::**  **:* *.**   ***
```

Supplementary Figure 5

Alignments of the N-terminus of H. sapiens MIER1 (Uniprot Q8N108) with D. melanogaster MIER1 (Uniprot A1Z6Z7) and C. elegans MIER1 (Uniprot P91437)

Supplementary Table S1. Excel file

Data related to Figure 5b. MS analysis of the MIER1:HDAC1:BAHD1 complex identifies C1QBP as a co-purifying protein.

Protein level data is contained in the "Protein groups" tab with complex components highlighted in yellow. Samples A-D correspond to gel bands containing HDAC1, MIER1, BAHD1 and C1QBP, respectively. Peptide data for C1QBP is included in the "C1QBP peptides" tab, with sequence coverage highlighted in red. All data is filtered to a 1% FDR at the protein and peptide level.

Supplementary Table S2. Excel file

Data related to Figure 5g. Quantitative MS analysis of histones co-purified with the MIER1(171-512):HDAC1:BAHD1:C1QBP reveals that the majority of H3K27 is di or tri-methylated. Protein level data is contained in the "Protein groups" tab. Identified acetyl, methyl, dimethyl and trimethyl lysine site identifications are listed in the relevant tabs. A summary of quantified levels of peptide (KSAPATGGVKKPHR) modification forms encompassing H3K27 is shown in the "H3K27 modification state" tab. All data is filtered to a 1% FDR at the protein and peptide level. Biological triplicates were performed for each group.
